# Growth Performance, Carcass and Meat Traits of Autochthonous *Arouquesa* Weaners Raised on Traditional and Improved Feeding Systems

**DOI:** 10.3390/ani12192501

**Published:** 2022-09-20

**Authors:** Laura Sacarrão-Birrento, Maria José Gomes, Severiano R. Silva, José A. Silva, Duarte Moreira, Raquel Vieira, Luis Mendes Ferreira, Pedro Pereira, André M. de Almeida, José Carlos Almeida, Carlos Venâncio

**Affiliations:** 1 LEAF—Linking Landscape, Environment, Agriculture and Food Research Center, Associated Laboratory TERRA, Instituto Superior de Agronomia, Universidade de Lisboa, Tapada da Ajuda, 1349-017 Lisbon, Portugal; 2 Veterinary and Animal Research Centre (CECAV) and Associate Laboratory of Animal and Veterinary Science (AL4AnimalS), University of Trás-os-Montes e Alto Douro, Quinta de Prados, 5000-801 Vila Real, Portugal; 3 Animal Science Department, University of Trás-os-Montes e Alto Douro, Quinta de Prados, 5000-801 Vila Real, Portugal; 4 Centre for Research and Technology of Agro-Environment and Biological Sciences (CITAB), University of Trás-os-Montes and Alto Douro, 5000-801 Vila Real, Portugal; 5 Cevargado—Alimentos Compostos, Unipessoal, Lda., Arcos, 4480-028 Vila do Conde, Portugal

**Keywords:** autochthonous *Arouquesa* breed, beef labeling, meat quality, mountain livestock, sustainable production

## Abstract

**Simple Summary:**

Animal production in mountain regions is changing and improvements to maintain autochthonous breeds and traditions should be defined. One of the strategies to add value and improve productivity is to produce meat with a PDO (protected denomination of origin) label. One example in Portugal is the *Arouquesa* PDO beef. This work aimed to compare different production systems and understand if they affect growth performance, carcass, and meat parameters for the *Arouquesa* PDO beef. Systems using supplementation had better results regarding live weight and average daily gain. The finishing period increased subcutaneous fat. The meat quality parameters differ in the improved production system with early weaning leading to lesser exudative and cooking losses. In conclusion, the traditional systems improved with practices, such as supplementation, can in turn improve meat production without affecting beef quality or PDO certification. This study demonstrates that improved production systems can advance the *Arouquesa* autochthonous breed production, while in turn maintaining the valued characteristics of a PDO product.

**Abstract:**

*Arouquesa* is an autochthonous bovine breed known for its *Arouquesa* PDO beef labeling. There are several production systems under the definition of PDO labeling. This study aimed to compare the effect of different production systems on carcass and meat traits for the *Arouquesa* breed. Two trials differing in diet and weaning age were conducted. The first trial included a TF group fed the traditional way and weaned at 9 months; a TF + S1 group, equal to TF, but with a starter supplement; and finally, a S1 + S2 group that was fed with a starter and a growth supplement and weaned at 5 months. The second trial was composed of a TF + S3 group fed like the TF + S1 group but reared until 12 months with a finishing supplement, and finally, the S3 group fed like the S1 + S2 group but reared until 12 months. In the first trial, the TF + S1 and S1 + S2 groups showed higher final live weight and average daily gain. In the second trial, we observed differences in the subcutaneous fat that was higher in the S3 group. Regarding meat traits, we observed differences in exudative and cooking losses in the first trial. In general, supplementation improved meat production without affecting meat quality parameters.

## 1. Introduction

Mountain livestock farming plays an important role in providing food, goods, and services to local populations [1]. In such systems and particularly in the European Union context, autochthonous ruminant breeds represent a valuable genetic resource and are mainly associated with sustainable production systems [2]. Additionally, local ruminant breeds have an important role in the maintenance of local traditions, the valorization of the local heritage, and gastronomy with the advantage of the high potential for adaptation and uniformity in the harsh local production conditions [3].

Over the last decades, the traditional mountain systems of the EU and the local breeds produced in such areas tended to decrease in importance and, in some cases, even disappear. This is due to socio-economic reasons that ultimately lead to the depopulation of rural marginal areas with severe consequences for food security and the environment [4]. In accordance, and to limit this problem, several programs have been promoted to support such production systems at the European, national, and local levels [5]. However, one of the interesting consequences of such programs was that smaller farms ceased their activities and there was a tendency toward increasing both farm areas and inventories, as well as grazing management extensification [6]. Furthermore, these farms stayed heavily dependent on subsidies to ensure their survival [7].

To change the future of traditional mountain systems, increase the sustainability, and add value to the local products, it is important to adapt new strategies and practices [8]. In Portugal, and over the last three decades, there has been a growing interest in traditional beef production systems. This is a result of an increase in environmental and gastronomical tourism activities [9], as well as a general increased demand for differentiated products that are generally linked with the protected denomination of origin (PDO) labels that ascertain consumers of a higher quality beef [10] and sustainable production practices.

**Figure 1:**
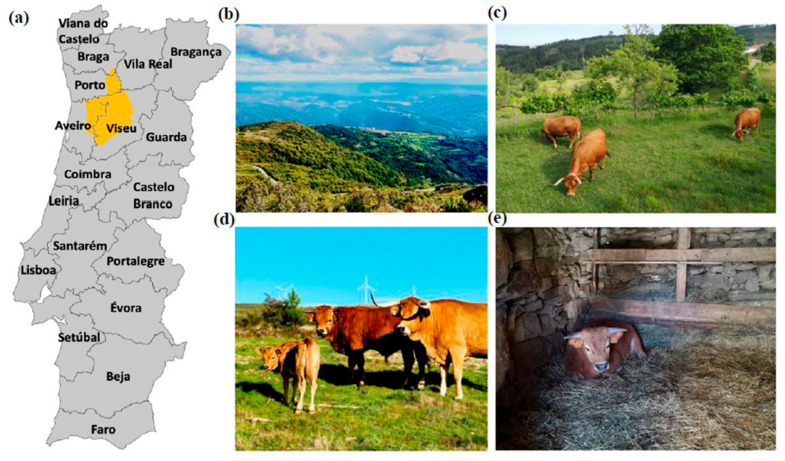
(**a**) Production area of *Arouquesa* breed; (**b**) *Montemuro* mountain landscape where is possible to observe the *Lameiros* pastures; (**c**) *Arouquesa* herd grazing in a *Lameiro*; (**d**) Animals in a *Lameiro* (From the left to the right, an *Arouquesa* calve, a weaner and a cow); (**e**) Sheds where the calves are kept.

In northern and central Portugal, the autochthonous *Arouquesa* cattle breed and the production systems associated with such animals are pertinent examples that fit in the above. There are 5119 adult animals registered in the *Arouquesa* herd book, namely 4893 cows and 226 bulls for a total of 1113 producers. Currently, the production area of the *Arouquesa* breed covers 3240 km^2^ [11] and is limited to 22 municipalities in mountainous regions in the north between the valleys of the Vouga and Douro rivers (Figure 1a) [12]. The production system is traditionally based on a subsistence agriculture system where a herd rarely exceeds five animals per farmer [13]. The traditional feeding system is characterized by highland grazing. Animals use spontaneous pastures that include several shrub and grass species such as heather (*Calluna vulgaris*) or gorse (*Ulex europaeus*), supplementation with green forage produced on non-irrigated land during a significant part of the year, and local *Lameiros* pastures (Figure 1b–d). *Lameiros* are permanent pastures of natural grassland on the slopes of mountains with run-off irrigation and overflow that are characteristic of northern and central Portugal [14]. Grazing takes place in the daytime and, since the availability and quality of pastures are not stable throughout the year, supplementary feed such as hay and straw can be provided during the night in rudimentary stone barns and sheds that are often used to house these animals (Figure 1e). At the end of spring, the animals stop grazing in the *Lameiros* pastures so that the grass can regrow to produce hay in the summer. Calves are kept indoors in the barns and sheds and suckling only occurs in the morning and at night as cows graze during the day [15]. Animals are traditionally weaned at 8–9 months of age and are then sold for slaughter. In such a system and at the age of 2–3 months, calves start receiving hay and/or straw, green fodder, and some concentrate feeds, usually ground maize (0.5 to 2 kg/head/day) [16]. Under such conditions, seasonal variations condition pasture growth and often lead to a nutritional restriction [17] that in turn leads to the mobilization of body reserves, severely affecting the animal’s growth ability, leading to body condition score fluctuations [18], overall affecting carcass uniformity and meat quality [19]. An improved production system is becoming more frequent. Such systems are frequent among farmers with larger inventories (between 30 and 50 cows). In such systems, cows are pasture-fed, calves are suckled in groups, and earlier weaning at 5 months is conducted, mostly due to a dropping decrease in milk production under natural pasture conditions. Subsequently, calves receive concentrate feed and forage until 8–9 months of age [16].

As the *Arouquesa* cattle breed has a PDO label—the *Arouquesa* PDO Beef—it has a very well-established set of production rules that farmers must abide. Nevertheless, it is important to improve the production strategies for adding value to such an important and iconic product, whilst maintaining the specifications of the PDO label, such as firm consistency, succulence, meat pale or light pink, and some aroma and flavor peculiar characteristics [20], and thus guarantee the uniformity of the product, fulfilling the high consumer expectations. To achieve such carcass uniformity, it is of the utmost importance to establish feed supplements that can balance the nutritional deficiencies of the diets available on the farms. Thus, the main objective of this work was to determine the effect of supplementation on growth, carcass traits, and meat quality performance of *Arouquesa* weaners produced under different feeding systems and when compared to the traditional system with the slaughter at nine months. Furthermore, another objective is to study these variables in two prolonged finishing systems with the slaughter at 12 months of age. The results are of major importance for beef production systems in mountainous areas of Portugal, the Iberian Peninsula, and the EU.

## 2. Materials and Methods

### 2.1. Systems and Experimental Design

In this work, two experiments were carried out using *Arouquesa* breed weaners originating from ten different farms located in the region of origin of the breed (Figure 1a). These farms were selected with the support of the National Association of *Arouquesa* Cattle Breeders (ANCRA). All farms have the same sanitary protocol. An experienced veterinarian regularly checked all the calves/weaners involved in this study. Animals that had health issues were not included in the study.

**Figure 2:**
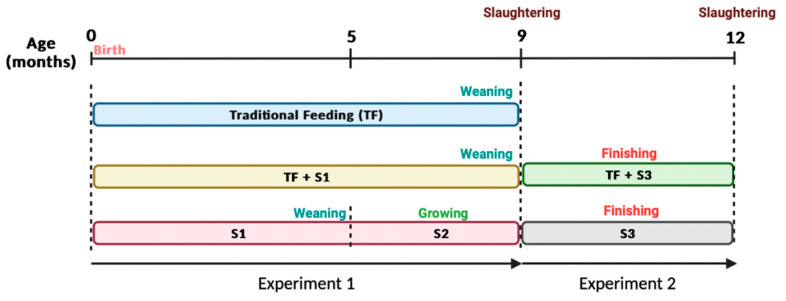
Study design for experiment 1 and experiment 2. Experiment 1 was composed of three groups: TF, animals fed with traditional feeding, weaned, and slaughtered at 9 months; TF + S1, animals were produced in the same way as the TF group but supplemented with a starter supplement (S1); and S1 + S2, fed with S1 until weaning at 5 months and with a growth supplement until slaughter at 9 months. Experiment 2 had two groups: TF + S3, animals weaned by the traditional way and fed with a finishing supplement (S3); and S3, animals finishing with the S3 supplement.

In experiment 1, 60 male weaners were evaluated for growth according to three feeding systems: the traditional feeding (TF), in which animals are weaned and slaughtered at 9 months, receiving hay and ground maize at 3 months of age; the TF + S1, that corresponds to the traditional system but half of the ground maize is replaced by a starter concentrate feed (S1) and animals are weaned and slaughtered at 9 months; and the S1 + S2, corresponding to an improved production system in which the S1 concentrate entirely replaces the ground maize, and animals are submitted for early weaning at 5 months of age with short rearing and then reared with S2 growth concentrate until slaughter at 9 months of age. In all the feeding systems, animals were fed *ad libitum*. Experiment 1 is schematized in Figure 2. The animals in the TF, TF + S1, and S1 + S2 groups had an initial live weight of 144 ± 8.1 kg (*n* = 11), 128 ± 5.2 kg (*n* = 23), and 123 ± 6.9 kg (*n* = 26), respectively. At 9 months, the 11 animals from the TF group and the 13 and 15 animals, respectively, from the TF + S1 and S1 + S2 groups were slaughtered.

In experiment 2, and as described in Figure 2, 21 male weaners at 9 months of age were divided into two groups differing in the feeding system. The first group was the TF + S3 in which, after the traditional weaning at 9 months, the animals received a finishing concentrate feed (S3) until they were slaughtered at 12 months of age. The second group was termed S3. In the latter group, animals were early weaned with extended rearing and subsequently fed with concentrate S1 until weaning at 5 months, then reared with the concentrate feed S2 until 9 months, finally finishing with the S3 concentrate feed until they were slaughtered at 12 months of age. The animals in the TF + S3 and S3 groups had an initial live weight of 247 ± 5.1 kg (*n* = 10) and 221 ± 11.0 kg (*n* = 11), respectively.

The ingredients of the concentrate feeds (S1, S2, and S3) were maize, barley, wheat bran, soybean meal, carob meal, calcium carbonate, salt, and a premix, but combined in different proportions, according to a phase feeding program (e.g., starter, growing and finishing concentrate, S1, S2, and S3, respectively). The main plant species present in the meadow hay were *Lolium perenne*, *Holcus lanatus*, and *Bromus* sp. The chemical composition of feeds is shown in Table 1.

### 2.2. Animal Growth

The live body weight for each animal was recorded every two weeks over the experimental period and always 2 h before morning feeding, until they achieved the target age. Animals were weighted in a R10 scale (Cachapuz, Braga Portugal) with a 1000 kg capacity and a 0.5 kg accuracy.

Average daily gain (ADG) of each animal was the coefficient of the linear regression of weights on days of experiment (DOE):

yi = α + β × DOEi + εi, (1)

in which yi = weight of the animal in the ith observation, α = intercept of the regression equation corresponding to the initial weight, β = linear regression coefficient corresponding to ADG, DOTi = days of test for the ith observation, and εi = random error associated with each observation.

### 2.3. Ultrasound Measurements

The real-time ultrasound (RTU) technique was used to assess, in vivo, the level of the subcutaneous fat thickness (SF_RTU) and the depth of Longissimus thoracis et lumborum muscle (LMdepth_RTU). Before slaughter, the animals were scanned using an ultrasound scanner (A6, SonoScape Co., Ltd., Shanghai, China) with a linear probe of 5 MHz. The animals were individually restrained and held by the head in a squeeze chute to minimize movements and ensure they stood in a similar stance. Then, physical palpation of the lumbar region was performed to accurately ascertain the scanning site. The probe was perpendicular to the backbone over the second lumbar vertebra (L2). An ultrasound gel was used as a coupling medium. The ultrasound images were captured and saved on the scanner for later analysis. Afterwards, the images were transferred to a computer and analyzed for the SF_RTU and LMdepth_RTU measurements using the Fiji software (http://fiji.sc/Fiji, accessed on 20 May 2022, ImageJ 1.49u, National Institutes of Health, Bethesda, MD, USA). For SF_RTU measurements, an average of three depths was considered. This procedure allows overcoming variations in the thickness of the subcutaneous fat over the Longissimus thoracis et lumborum muscle.

### 2.4. Slaughter and Carcass Traits

Animals were slaughtered at a certified abattoir. After slaughter, carcasses were dressed, centrally split into two halves, and then chilled for 24 h at 2 °C. In addition, for each carcass, the lumbar cut from the first to the fifth vertebrae was obtained. This cut was refrigerated and transferred to the laboratory for subsequent analyses.

### 2.5. Lumbar Measurements

The lumbar cut was divided into small cuts for meat quality physiochemical traits. In the cut plane at the level of the second vertebra, images were captured using a digital camera with a 16 megapixel sensor. A scale was placed for tissue feature measurements in all cuts. Images were transferred to a computer and analyzed using Fiji software (ImageJ 1.49u, NIH, USA). For tissue measurements, the first step is to convert the pixels to mm, using the scale. Four measurements were determined for LM muscle (area, perimeter, major axis, and LM depth). Furthermore, the subcutaneous fat (SF) depth was determined. For this, SF thickness measurements were taken at three sites above the LM, and the average was calculated.

### 2.6. Post-Mortem Meat Quality Traits

The Longissimus thoracis et lumborum muscle was collected at 24 h, and packed and aged for 7 days at 4 °C. Both the pH and sarcomere length were measured at 24 h. Exudative losses, cooking losses, shear force, and color were measured after 7 days.

#### 2.6.1. pH

The pH was measured at 24 h (pH_24h_) with a penetration electrode accoupled to pH meter WTW 330i (Weilheim, Germany) after calibration with buffers of pH 4.01 and 7.00.

#### 2.6.2. Color

The LM surface color measurements were obtained with Minolta Chroma Meter CR-310 colorimeter (Osaka, Japan) and assessed using the L*, a*, and b* three-dimensional color space, defined by [21]. In this system, L*, a*, and b* represent the measurements of luminosity, red-green, and yellow-blue, respectively. The color was measured on the meat surface after 60 min of blooming by placing the samples in trays covered with polyethylene film at 4 °C [22]. The colorimeter was calibrated before the usage with a standard white ceramic plate and D_65_ illuminant was used.

#### 2.6.3. Exudative Losses

Exudative losses (EL) were determined by the difference between the initial weight (W_i_) and the final weight (W_f_) at day 7 after 6 days of storage vacuum-packed at 4 °C, and were expressed as a percentage of the initial weight [23]:

Exudative losses (%) = [(W_i_ − W_f_)/W_i_] × 100. (2)

#### 2.6.4. Cooking Losses

Samples of about 90–100 g were weighed and individually placed in a polyethylene bag and then placed in a Digiterm 100 water bath (JP Selecta, Barcelona, Spain) at 80 °C until an internal temperature of 71 °C was reached, monitored with a thermocouple. Subsequently, each sample was placed in ice water until the internal temperature reached 15 °C. It was then stored for about 24 h at 4 °C. The meat was removed from the bag, carefully dried, and weighed, thus obtaining the final weight. Cooking losses (CL) were determined by the difference between the initial (W_i_) and final (W_f_) weights after cooking, and were expressed as a percentage of the initial weight [23]:

Cooking losses (%) = [(W_i_ − W_f_)/W_i_] × 100. (3)

These samples were subsequently packaged and stored at 4 °C to be used in shear force determination.

#### 2.6.5. Sarcomere Length

Sarcomere length was evaluated at 24 h using the method described in [24]. Briefly, approximately 2 g of LM was cut with a scalpel in small portions to which 30 mL of a cold 0.25 M sucrose solution was added, and subsequently homogenized at a slow speed for 60 s with the Ultra-Turrax T25B (Kika Labortechnik, Staufen, Germany). A drop of the homogenate was placed on a slide, using a Pasteur pipette, and it was covered with a coverslip, having been observed under the Nikon Labophot-2 (Nikon, Tokyo, Japan) optical microscope in phase 1, with the 40 × objective with attached camera. The length of 10 consecutive sarcomeres was measured, and approximately 15 groups of 10 sarcomeres per sample were measured using the image analysis software Matrox Inspector 4.1 (Matrox Electronic Systems Ltd., Dorval, QC, Canada). The average length of the sample sarcomeres was subsequently determined.

#### 2.6.6. Shear Force

Meat samples used to determine the cooking losses were removed from the refrigerator and were cut into cuboid shape sub-samples (6 to 8) of 1 cm^2^ cross-section and 3–4 cm in length with muscle fibers parallel to the length of the cuboid. After room temperature equilibrium, the sub-samples were placed with fibers perpendicular to the direction of a Warner–Bratzler rectangular hole probe coupled to a TA.XT.plus texturometer (Stable Micro Systems, Godalming, UK), with a load cell of 30 kg.f., blade velocity set to 200 mm/min, and trigger force of 5 g. Maximum shear force values (SF) were recorded and the values were expressed in N/cm^2^.

### 2.7. Statistical Analysis

An analysis of variance (ANOVA) was performed to study the effect of the feeding system on final live weight, ADG, ultrasound measurements, carcass weight, carcass yield, lumbar measurements, and meat quality traits. ANOVA analyses were performed for animals slaughtered at age 9 months (experiment 1) and 12 months (experiment 2). For both experiments, initial live weight was included as a covariate adjustment term in the analysis of AGD, final live weight, RTU measurements, and CW and carcass yield. For models pertaining to meat traits (LM measurements and meat quality), the carcass weight was included as a covariate. The LSMeans Differences Student’s multiple comparison test was used to compare the least square means. Data were analyzed by JMP 15 (SAS Institute Inc., Cary, NC, USA). Significance was declared when *p* < 0.05.

### 2.8. Animal Welfare Disclaimer

All conditions and procedures performed with the animals were recognized as common animal husbandry practices that took place according to Portuguese (Decreto-Lei 113/2013) and EU (directive 2010/63/EU) legislation for animal experimentation and welfare. The procedures were carried out upon approval by the University of Trás-os-Montes e Alto Douro animal experimentation committee under reference 1507-e-DZ-2020.

## 3. Results and Discussion

### 3.1. Live Weight, Ultrasound Traits, Carcass Weight and Lumbar Measurements

The least-squares analyses were used to investigate the effect of the feeding system on live weight, ultrasound measurements, carcass weight and yield, and lumbar measurements (Table 2 and Table 3). Although both experiments were performed on the field and the ranges of initial live weight (LW) within the group were high, the initial LW between groups was not statistically different (*p* = 0.273); it can be ascertained that such variability levels did not condition the results.

In experiment 1, the feeding system had a significant effect (*p* < 0.05) on ADG and final LW. Animals fed TF + S1 and S1 + S2 achieved a similar ADG (1006.3 and 1004.2 g/day, respectively), representing a 16% increase (*p* < 0.05) when compared with animals submitted to traditional feeding (ADG = 867.2 g/day). The increased ADG resulted in approximately 14% heavier (*p* < 0.05) animals at the time of weaning in both groups (LW = 272.6 and 273.4 kg for TF + S1 and S1 + S2, respectively vs. 240.2 kg for TF). Average carcass weights (CW) were numerically heavier in these two treatments (approximately 14 kg) when compared with animals in the traditional system, however, such differences were not significant (*p* = 0.266).

In beef cattle, milk is generally not sufficient to satisfy the calves’ nutritional requirements when they are about 3 months of age [25]. Indeed, and under range conditions, the quality and quantity of pasture available for cows are not constant throughout the year, and in some situations leads to nutritional restrictions and consequently mobilization of body reserves [17]. Previous studies reported that milk yield and content (fat, protein, and lactose) are linearly affected by the cow’s postpartum body condition [26]. Noya et al. [27] showed an interaction between maternal nutrition and weaning and slaughter as well as carcass weights, so it is essential to supplement the animals with concentrate to compensate for nutritional deficiencies. For these reasons, at such ages, calves become increasingly dependent on solid feeds; thus, strategic supplementation can increase animal performance. Traditional supplementation of *Arouquesa* weaners relies on feeds locally produced by the farmer (usually pasture hay and maize grain). Maize is a rich energy source, but with low crude protein content. As shown in Table 1, the S1 supplement has higher values of PDIE (11.7 vs. 8.4%) and PDIN (13.5 vs. 6.3%) than maize. Thus, it is expected that by replacing half of maize with the S1 supplement, the ruminal microflora growth will improve, as well as the digestion of organic matter in the rumen [28]. Consequently, a greater absorption of volatile fatty acids in the rumen and flow of microbial protein to the intestine are also expected. In fact, according to [28], a higher digestible microbial protein flow to the intestine must have occurred, but, regarding the higher PDIA content of the S1 supplement relative to maize (7.3% vs. 4.6%; Table 1), also a higher flow of protein of feed origin (PDIA), resulting in a higher supply of amino acids to the animals. Ultimately, improved energy and amino acids uptake could allow better growth performance [29].

Early weaning and feeding of a phase-feeding concentrate (i.e., S1 and S2 concentrates) allowed the same responses regarding ADG, LW, and CW to be observed for animals weaned at 9 months, but receiving a supplement (S1) in addition to the traditionally offered feed (maize). Despite the anticipation of weaning age, the replacement of milk by the S2 supplement seems to be equally adequate to satisfy the nutritional needs of calves, since it allowed the achievement of identical productive responses. Other studies reported that animals that have not been weaned show better results in productive traits than early weaned animals [30,31]. However, this last group can show higher ADG in the finishing period due to compensatory growth [30].

To the best of our knowledge, there are no studies available regarding the introduction of concentrates on traditional feeding systems in Portuguese mountain autochthonous breeds. Nevertheless, they are long available for other European continental beef breeds, such as the Charolais X Friesian crosses or Southern Iberian breeds like the *Alentejana* [28,32].

We observed no differences between feeding systems for RTU and lumbar measurements (*p* > 0.05). The pattern of variation for the RTU measurements and the equivalent measurements obtained directly from the LM in the cut is similar. This result supports the significant (*p* < 0.01) correlation between those variables (r values between 0.69 and 0.96 for LM depth and 0.87 and 0.95 for SF; data not shown). These results show that the RTU technique helps obtain in vivo information related to carcass traits, as discussed in previous works on RTU for different meat species [33,34,35,36]. Specifically, for cattle, there are multiple studies in which the RTU technique was used to estimate carcass characteristics [37,38,39]. As such, it seeks to fulfill the need to use an instrumental system that can determine SF depth in vivo rapidly, precisely, and accurately. Furthermore, ultrasound data are used for genetic evaluations for relevant carcass traits [39,40], determining an optimal finishing point [41] and ultimately moving toward a value-based marketing system [33]. These last two aspects are relevant to creating value for carcass and meat of autochthonous breeds [10,14], such as the one analyzed in the present work.

The CW and lumbar measurements were not different (*p* > 0.05) between groups. These results were not expected since most of the studies reported higher carcass weights when increasing the concentrate levels [28,42]. The muscle area and subcutaneous fat tend to be higher in grass-fed animals when compared with supplemented animals, likely due to higher efficiency in the conversion of feed into weight gain [42]. The *Arouquesa* production system is quite different from most standard beef production systems, as the animals are weaned and slaughtered at 9 months. Accordingly, in other studies, early weaned animals (3 months) do not show differences regarding carcass traits when compared with animals weaned at 9 months [43,44].

Table 3 shows the least square means and standard errors for LW, ultrasound measurements, carcass weight, and lumbar measurements for steers from experiment 2. The feeding system did not affect final LW (*p* = 0.688), but a trend was observed for a higher growth rate of animals on S3 treatment. However, animals on TF+S3 presented a carcass weight of 12 kg heavier. The difference, however, was not significant (*p* = 0.139). A trend for higher carcass yields (54.6 vs. 50.4%) was registered.

There were no significant differences (*p* > 0.05) for LMdepth_RTU (mm). However, SF-RTU and SF were higher (*p* < 0.05) in animals in the S3 feeding system. It has been demonstrated that early weaned animals, when fed the S3 supplement (which provides a higher protein:energy ratio than maize plus S3) from 9 to 12 months of age, could achieve similar LW, albeit with carcasses with higher subcutaneous fat depth. Wolcott et al. [45] compared the subcutaneous fat between animals fed with high energy and animals fed with low energy and observed similar results. These observations may be associated with a compensatory growth in the S3 feeding system. Wright and co-authors [46] have long reported that compensatory growth in steers following a food restriction period and a discontinued growth showed an initial stage characterized mostly by muscle and protein deposit and a second phase where fat deposition predominates.

### 3.2. Meat Quality Traits

The values of least square mean and standard errors for meat quality traits for animals on the TF, TF + S1, and S1 + S2 feeding systems (Trial 1) are presented in Table 4.

No effects of feeding systems on meat pH_24_ were observed (*p* > 0.05). Interestingly, we can observe that pH values are higher than those described in other studies. Indeed [47], for Angus steers finished with legume-grass pasture, legume-grass pasture with whole corn grain, and grain-only diet, meat pH values of 5.61, 5.63, and 5.62, respectively, were presented, and as in our study, the values did not differ between groups. Fruet et al. [48] reported an average pH_24h_ of 5.74 for crossbreds and *Mirandesa*, a Portuguese mountain breed bearing some similarities to the *Arouquesa* breed. These results may be due to the animals’ age as younger animals tend to have lower muscle glycogen contents and pH drop rate and are more susceptible to stress, which causes higher pH values [48]. Additionally, we can hypothesize that some factors may have contributed to the high pH values observed, thus conditioning the potential effect of the farming system on meat quality traits. The animals from the *Arouquesa* breed are raised by small-scale producers in mountain regions with high dispersion and poor road access. These animals have limited contact with people or other animals. During transport to the abattoir, carried out the day before slaughter, animals are collected from different farms so that the duration of transport and the mixing of animals from different farms may contribute to the depletion of glycogen reserves. Abubakar et al. [49] also showed that some factors during transport, such as its length and stocking density, will affect meat pH and glycogen reserves. Thus, if transport duration increases, the pH tends to be higher and the glycogen lower thus explaining our results. In accordance, [50] showed that the presence of stress hormones due to transportation tends to increase the pH and 80% of the studied animals had a pH higher than 5.8.

For the color measurements, no differences were observed between groups (*p* > 0.05). For other breeds produced in mountain systems, such as Angus, Charolais, Norwegian Red, and Simmental, there were reported values of luminosity, red-green, and yellow-blue of 41.5, 19.7, and 5.1, respectively [51]. Other studies also did not observe differences between animals fed forage or grain in meat color measurements [46,52]. The color of fresh meat is an important quality attribute that may influence consumer preference during purchasing, as the bright cherry-red color tends to be preferred [53]. The lower L* values found in this work may be explained by the high pH_24h_ values, resulting in dark meat [54,55].

Exudative and cooking losses were affected by the feeding system (*p* < 0.05), contrary to sarcomere length and shear force (*p* > 0.05). Ijaz et al. [56] studied the effects of concentrate inclusion in grass silage-based diets for steers. They concluded that diet does not influence sarcomere length, cooking loss, and shear force. Sarcomere length after rigor mortis is influenced by several factors, such as the rate of pH decline, mechanical restraints, and the cooling rate of muscles, and can play an important role in meat tenderness [57]. The least-square mean values of sarcomere length are lower than 1.98 μm, reported by [58], or 1.85 μm, reported by [59], but similar to the values of 1.7 μm, reported by other authors [60]. Shear force was found to be increased in *Arouquesa* beef when weaning occurs at 5 months compared with weaning at 9 months in a study that also evaluated the sensory characteristics in samples with pH_24h_ mean values of 5.6 [61]. The lower values found in this work can be due to the higher pH values which can lead to a decrease in shear force [62].

Several works refer to a positive relationship between the ultimate pH and meat water holding capacity [62,63]. The significantly lower exudative and cooking losses found in the S1 + S2 group may be explained by the higher pH_24h_ found in this group, despite the pH_24h_ differences between groups not being significant. Hornick et al. [64] observed in bulls an increase of pH following a food restriction period with a trend to lower drip losses when compared with carcasses of continuous growth. Table 5 shows the values of least square means and standard errors for meat quality traits for steers from Traditional + S3 and S3 finishing.

No differences were observed between the finishing system and meat quality traits. The values of pH, despite being elevated, were closer to the normal values, probably because the animals were slaughtered at 12 months of age, and as such, were older than the animals of the first trial. Color measurements were not affected (*p* > 0.05). In addition, in other studies where the animals were weaned at different ages, there were no recorded differences in muscle color [31]. Finally, the EL, CL, CS, and SF also did not differ between groups (*p* > 0.05). Regarding the latter aspect, a finishing period can be recommendable in the improved farming systems with early weaning without compromising meat quality.

*Arouquesa* beef is a PDO product, so it has label specifications and production standards that must be fulfilled. By observing that supplementation does not affect the meat quality traits, we can consider such improved feeding systems that in turn will improve animals’ growth and carcass traits. However, the high meat pH_24h_ values can contribute to masking the potential effect of the farming system on meat quality traits, and possible causes for this observation need to be evaluated and ruled out. Furthermore, despite the fact that the aim is to slaughter the calves of the improved production system at nine months of age, they need different support in the early life stage or in the weaning period, with the best evaluation required. Moreover, the implication of these production systems on the distribution of carcass fat depots as well as meat composition needs to be addressed in future studies.

## 4. Conclusions

In this work, we describe for the first time the influence of the production system on growth and meat traits for one of the least studied mountain autochthonous cattle breeds in Portugal, the *Arouquesa* breed. The results may be extrapolated to other understudied and related mountain breeds in Portugal and the NW Iberian Peninsula subjected to similar production systems. Overall, the production system according to different feeding, weaning, and slaughtering ages influenced the live weight gains, carcass traits, and lumbar measurements but generally did not affect the meat quality parameters in the *Arouquesa* weaners although the high pH_24h_ may have conditioned the potential effect of the farming system on meat quality traits. Differences in the values of exudative and cooking losses may derive from factors other than the effect of the feeding regime that must be evaluated. Furthermore, the tested system preserved the meat quality compared with the traditional system of *Arouquesa* PDO beef. Essentially, the study showed that when introducing novel systems in this breed with added supplementation to the traditional feeding, it will improve beef production without affecting the PDO label specifications.

## Figures and Tables

**Table 1:** Chemical composition and energy and protein values of feeds.

		Meadow Hay	Ground Maize	S1	S2	S3
Chemical composition ^1^						
Dry matter	%	84.31	84.49	87.50	87.45	87.43
Ash	%	5.70	1.61	5.46	4.62	4.39
Crude protein	%	8.18	9.08	18.56	16.60	15.57
Crude fat	%	nd	nd	3.33	3.80	4.21
Crude fiber	%	nd	nd	4.32	4.84	4.77
NDF	%	69.10	20.54	15.08	14.59	14.10
ADF	%	41.86	4.23	5.31	5.88	5.58
ADL		6.89	0.86	nd	nd	nd
Starch	%	nd	nd	35.48	40.57	43.87
Calcium	%	nd	nd	0.64	0.42	0.40
Phosphorus	%	nd	nd	0.46	0.40	0.34
Sodium	%	nd	nd	0.20	0.20	0.20
Chlorine	%	nd	nd	0.39	0.38	0.38
Magnesium	%	nd	nd	0.21	0.22	0.27
Potassium	%	nd	nd	0.97	0.81	0.73
Energy and protein values ^2^						
UFV	UF·kg^−1^	0.57	1.06	0.98	1.01	1.03
PDIA	%	2.6	4.6	7.3	6.5	6.1
PDIE	%	7.1	8.4	11.7	10.9	10.5
PDIN	%	5.6	6.3	13.5	12.0	11.2

^1^ NDF = neutral detergent fibre; ADF = acid detergent fibre; ADL = lignin acid detergent. ^2^ UFV = feed unit for meat production, PDIA = protein digestible in the intestine (PDI) of feed origin; PDIE = PDI when energy is the limiting factor for rumen microbial activity; PDIN = PDI when nitrogen is the limiting factor for rumen microbial activity.

**Table 2:** Least square means and standard errors (in parenthesis) for live weight, ADG, ultrasound measurements, carcass weight, carcass yield, and lumbar measurements for steers from TF, T + S1, and S1 + S2 feeding systems (Experiment 1).

Traits	Feeding System			*p*
	TF (*n* = 11)	TF + S1 (*n* = 13)	S1 + S2 (*n* = 15)	
LW final (kg)	240.2 ^b^ (8.9)	272.6 ^a^ (6.9)	273.4 ^a^ (7.5)	0.013
ADG (g·day^−1^)	867.2 ^b^ (64.9)	1006.3 ^a^ (118.0)	1004.2 ^a^ (178.0)	0.027
Ultrasound				
LMdepth_RTU (mm)	59.8 (2.8)	58.9 (4.9)	56.8 (6.0)	0.680
SF_RTU (mm)	5.2 (0.74)	6.9 (0.63)	5.8 (0.62)	0.145
CW (kg)	122.2 (19.3)	136.1 (21.6)	136.5 (16.4)	0.266
Carcass yield (%)	51.0 (6.9)	50.2 (9.9)	49.9 (4.7)	0.911
Lumbar measurements				
Area (mm^2^)	4617.7 (263.5)	4822.1 (271.5)	4422.2 (226.6)	0.541
Perimeter (mm)	287.8 (9.9)	295.8 (10.2)	283.4 (8.5)	0.660
Major (mm)	107.3 (4.1)	111.9 (4.2)	107.0 (3.5)	0.659
LMdepth (mm)	54.5 (1.5)	54.5 (1.6)	52.1 (1.3)	0.402
SF (mm)	4.65 (0.42)	5.87 (0.43)	5.13 (0.36)	0.148

^a,b^ Rows with different superscripts indicate statistical differences (*p* < 0.05); TF—traditional system in which the animals are fed with hay and ground maize and are weaned and slaughtered at 9 months; TF + S1—corresponds to the TF but half of the ground maize is replaced by the S1 (starter supplement); S1 + S2—the ground maize is totally replaced by the S1 until weaning at 5 months and then the animals are reared with the S2 (growth supplement) until slaughtering at 9 months; LW—live weight; ADG—average daily gain; SF_RTU—subcutaneous fat thickness obtained with ultrasound; LMdepth_RTU—depth of Longissimus thoracis et lumborum muscle obtained with ultrasound; CW—carcass weight; LMdepth—depth of Longissimus thoracis et lumborum muscle; SF—subcutaneous fat depth.

**Table 3:** Least square means and standard errors (in parenthesis) for live weight, ultrasound measurements, carcass weight, and lumbar measurements for steers from TF+S3 and S3 finishing systems (Experiment 2).

Traits	Feeding System		*p*
	TF + S3 (*n* = 10)	S3 (*n* = 11)	
LW final (kg)	307.3 (6.1)	310.8 (4.9)	0.688
ADG (gday-1)	937.368.1)	1122.2 (54.9)	0.074
Ultrasound			
LMdepth_RTU (mm)	65.0 (3.3)	64.1 (3.8)	0.872
SF_RTU (mm)	5.8 ^b^ (0.38)	8.3 ^a^ (0.43)	0.002
CW (kg)	167.7 (5.5)	155.4 (4.5)	0.139
Carcass yield (%)	54.6 (1.6)	50.4 (1.3)	0.077
Lumbar measurements			
Area (mm^2^)	5352.1 (235.8)	5239.8 (273.2)	0.790
Perimeter (mm)	306.9 (6.7)	319.4 (7.7)	0.304
Major (mm)	116.5 (2.2)	114.4 (2.6)	0.604
LMdepth (mm)	57.7 (2.0)	60.5 (2.3)	0.449
SF (mm)	5.5 ^b^ (0.41)	7.1 ^a^ (0.47)	0.037

^a,b^ Rows with different superscripts indicate statistical differences (*p* < 0.05); TF + S3—the animals are fed by the traditional system and weaned at 9 months, but receive a finishing concentrate (S3) until the slaughter at 12 months; S3—animals are fed with S1 (starter supplement) until weaning at 5 months, then fed with S2 (growth supplement) until 9 months and finally reared with S3 until slaughtering at 12 months; LW—live weight; ADG—average daily gain; SF_RTU—subcutaneous fat thickness obtained with ultrasound; LMdepth_RTU—depth of Longissimus thoracis et lumborum muscle obtained with ultrasound; CW—carcass weight; LMdepth—depth of Longissimus thoracis et lumborum muscle; SF—subcutaneous fat depth.

**Table 4:** Least square means and standard errors (in parenthesis) for meat quality traits for steers from TF, TF+S1, and S1+S2 feeding systems (Experiment 1).

Traits	Feeding System			*p*
	TF (*n* = 11)	TF + S1 (*n* = 13)	S1 + S2 (*n* = 15)	
pH_24h_	6.29 (0.13)	6.11 (0.12)	6.45 (0.10)	0.101
L*	37.9 (1.4)	38.3 (1.2)	35.5 (1.0)	0.156
a*	23.1 (2.4)	23.2 (2.5)	22.9 (2.8)	0.903
b*	5.87 (0.60)	6.18 (0.52)	4.66 (0.45)	0.079
EL (%)	2.31 ^a^ (0.33)	1.85 ^a^ (0.29)	0.97 ^b^ (0.25)	0.006
CL (%)	13.5 ^a^ (1.5)	14.0 ^a^ (1.3)	10.0 ^b^ (1.1)	0.049
CS (µm)	1.69 (0.053)	1.63 (0.047)	1.71 (0.041)	0.493
SF (N/cm^2^)	47.1 (8.7)	52.9 (7.6)	51.5 (6.6)	0.878

^a,b^ Rows with different superscripts indicate statistical differences (*p* < 0.05); TF—traditional system in which the animals are fed with hay and ground maize and are weaned and slaughtered at 9 months; TF + S1—corresponds to the TF but half of the ground maize is replaced by the S1 (starter supplement); S1 + S2—the ground maize is totally replaced by the S1 until weaning at 5 months and then the animals are reared with the S2 (growth supplement) until slaughtering at 9 months; pH_24_h—pH measured at 24 h post mortem; L*—luminosity measured after 60 min of blooming; a*—red-green measured after 60 min of blooming; b*—yellow-blue measured after 60 min of blooming; EL—exudative losses measured at day 7 post mortem; CL—cooking losses measured at day 7 post mortem; CS—sarcomere length; SF—Warner–Bratzler shear force measured at day 7 post mortem.

**Table 5:** Least square means and standard errors (in parenthesis) for meat quality traits for steers from TF + S3 and S3 finishing systems.

Traits	Feeding System		*p*
	TF + S3 (*n* = 6 *)	S3 (*n* = 11)	
pH_24h_	6.2 (0.24)	5.8 (0.14)	0.268
L*	36.9 (1.9)	39.4 (1.1)	0.323
a*	23.3 (1.4)	24.8 (0.82)	0.408
b*	6.5 (1.2)	7.7 (0.68)	0.426
EL (%)	2.0 (0.94)	2.9 (0.53)	0.426
CL (%)	16.7 (3.1)	18.1 (1.8)	0.724
CS (µm)	1.67 (0.04)	1.75 (0.02)	0.167
SF (N/cm^2^)	50.8 (10.9)	67.4 (6.2)	0.237

* Due to cooling problems, four of the ten samples were not used; TF + S3—the animals are fed by the traditional system and weaned at 9 months, but receive a finishing concentrate (S3) until the slaughter at 12 months; S3—animals are fed with S1 (starter supplement) until weaning at 5 months, then fed with S2 (growth supplement) until 9 months and finally reared with S3 until slaughtering at 12 months; pH_24h_—pH measured at 24 h post mortem; L*—luminosity measured after 60 min of blooming; a*—red-green measured after 60 min of blooming; b*—yellow-blue measured after 60 min of blooming; EL—exudative losses measured at day 7 post mortem; CL—cooking losses measured at day 7 post mortem; CS—sarcomere length; SF—Warner–Bratzler shear force measured at day 7 post mortem.

## Data Availability

The data presented in this study are available on request from the corresponding author. The data are not publicly available to preserve privacy of the data.
